# Oxidative Stress Mediated by Macrophages Promotes Angiogenesis and Early Development of Endometriosis

**DOI:** 10.3390/antiox15020159

**Published:** 2026-01-23

**Authors:** Gene Chi Wai Man, Astrid Borchert, Tao Zhang, Sze Wan Hung, Hartmut Kühn, Chi Chiu Wang

**Affiliations:** 1 Department of Obstetrics and Gynaecology, The Faculty of Medicine, The Chinese University of Hong Kong, Prince of Wales Hospital, Hong Kong SAR, China; geneman@cuhk.edu.hk (G.C.W.M.); taozhang@cuhk.edu.hk (T.Z.); szewanhung@cuhk.edu.hk (S.W.H.); 2 Department of Orthopaedics and Traumatology, The Faculty of Medicine, The Chinese University of Hong Kong, Prince of Wales Hospital, Hong Kong SAR, China; 3 Department of Biochemistry, Charité-Universitätsmedizin Berlin, Charitéplatz 1, 10117 Berlin, Germany; astrid.borchert@charite.de (A.B.); hartmut.kuehn@charite.de (H.K.); 4 Li Ka Shing Institute of Health Sciences, The Faculty of Medicine, The Chinese University of Hong Kong, Prince of Wales Hospital, Hong Kong SAR, China; 5 School of Biomedical Sciences, Faculty of Medicine, The Chinese University of Hong Kong, Hong Kong SAR, China

**Keywords:** endometriosis, oxidative stress, macrophage, angiogenesis, HIF-1α, PX-478

## Abstract

Endometriosis is a hormone-dependent gynecological disease manifested by cyclic pelvic pain and female infertility. Although many studies have shown that neoangiogenesis plays an essential role in the development of early endometriosis, the underlying pathophysiological mechanisms remain unclear. Recent evidence suggests that macrophages play an important role in the pathogenesis of endometriosis and that the hypoxia-inducible factor-1alpha (HIF-1α) may be involved, but when and how are largely unknown. Herein, we explore the role of macrophages in the early development of endometriosis using an in vivo subcutaneous implantation murine model. Upon depletion of macrophages, the subcutaneous injection of syngeneic endometrial material resulted in significant reduction in oxidative stress, endometriotic lesion size, and neovascularization. Likewise, inactivation of the lipid peroxidative gene Alox15 induced similar reduction in oxidative stress, lesion growth, and angiogenesis. Since HIF-1α is an important trigger of neoangiogenesis, we further administered a HIF-1α-specific inhibitor (PX-478) to our endometriotic model and further confirmed the same effects on the lesions. Taken together, these data suggest that an intact Alox15 pathway and HIF-1α signaling may play important roles in the macrophage-mediated oxidative stress and neovascularization of endometriosis in the early stages, suggesting anti-inflammation and antioxidation as potential therapeutic targets for the development of endometriosis.

## 1. Introduction

Endometriosis is a frequently occurring gynecological disorder that is characterized by the presence of tissue comprising endometrial glands and stroma outside the uterine cavity. It affects approximately 5–10% of women at the reproductive age [1]. It is one of the major causes of chronic pelvic pain, menstrual disorder, and female infertility. Although there remain multiple theories on the pathogenesis of endometriosis, Sampson’s concept of retrograde menstruation has widely been accepted [2]. However, retrograde menstruation occurs in 76% of women, but only 10–24% of them develop endometriosis [3]. It has been hypothesized that there exist defects in the immune system of women with endometriosis, which is either unable to recognize endometrial cells in ectopic environments or to initiate a proper immune response to remove these cells [4]. A functional immune system should be capable of identifying endometrial cells and preventing their implantation in other tissues.

Early development of endometriosis can be characterized by four stages, which include inflammation, immune evasion, tissue remodeling, and angiogenesis [5]. Various immune cells, including macrophages, natural killer (NK) cells, and regulatory T-cells (Tregs), are important for the development of endometriosis [6], but macrophages may play a dual role. They can eliminate endometriotic cells from ectopic tissue as part of a proper immune response, but they may also foster the growth of endometriotic lesions [7]. The macrophage-mediated anti-endometriotic immune response is rather complex and includes the release of pro- and anti-inflammatory mediators [8].

Numerous reports have previously shown that macrophages infiltrate early endometriotic lesions [9,10]. In patients suffering from endometriosis, there are increased numbers of macrophages and other leukocytes in and around endometrial implants and in the peritoneal fluid. Macrophage polarization is important for vascularization of endometriotic lesions [11,12] and for the infiltrating capabilities of endometrial cells [13]. In fact, neovascularization is an essential process for the survival of endometriosis implants. In addition, macrophages further deliver signals to limit apoptosis of endometriotic cells and facilitate local infiltration and metastatic spreading [14].

As in cancer, neoangiogenesis plays a critical role in the progression of endometriosis. Endometriotic lesions need extensive neovascularization to provide oxygen and nutrients for lesion growth, and oxidative stress plays an important role in inflammation and neovascularization [15]. In endometriosis, oxidative stress, defined as an imbalance between the formation of reactive oxygen species (ROS) and the antioxidative capacity, induces local inflammation in and around endometriotic implants [16]. Moreover, elevated levels of oxidative stress and an impaired antioxidative capacity have been reported in patients suffering from endometriosis [17,18,19]. The formation of ROS is essential for the proper function of macrophages and other phagocytosing immune cells such as neutrophils and B-cells. Moreover, macrophages contribute to the formation of oxidative stress and ROS during the pathogenesis of inflammatory diseases, such as stroke, myocardial infarction, chronic heart failure, diabetes, circulatory shock, cancer, and neurodegenerative disorders [20]. For the pathogenesis of endometriosis, it has been speculated that activated macrophages in the peritoneal cavity generate oxidative stress and thus promote the formation and growth of endometriotic lesions. However, no direct data are available, and the underlying molecular pathway involved in the formation of oxidative stress and how it promotes neoangiogenesis remains unclear.

Herein, in this study, we studied the mechanism of macrophages on angiogenesis in the early phase of the development of endometriotic lesions. We hypothesize that increased macrophage infiltration into the endometriotic lesions induces oxidative stress and ROS, which is essential for lesional endometriosis neovascularization.

## 2. Materials and Methods

### 2.1. Animal Experiments

All experiments were approved by the Animal Experimentation Ethics Committee (12/004/DRG) of the CUHK and by the Animal Care Committee (Landesamt für Gesundheit und Soziales, Berlin, Germany, permission number G 0297/12). The experiments were performed in accordance with institutional guidelines. Female C57BL/6J mice, transgenic UbC-Luc^+/+^ mice, and female Alox15^-/-^ mice (15-lipoxygenase knockout mice, 5–6 weeks old, 16–20 g body weight) were used in this study. Wild-type C57BL/6J and transgenic UbC-Luc^+/+^ female mice were obtained from the Laboratory Animal Services Center of the Chinese University of Hong Kong. A colony of Alox15^-/-^ mice [21] was bred in the animal house of Charité–Universitätsmedizin, Berlin, and female individuals of this colony were used for these experiments. Alox15 deficiency in these animals was tested by ex vivo Alox15 activity measurements using peritoneal lavage cells as the enzyme source [22]. Mice were housed in plastic cages at a temperature of 20–22 °C, a humidity of 30–50%, and a 12 h/12 h light/dark cycle. Autoclaved chow pellets and autoclaved tap water were provided *ad libitum*.

### 2.2. Ovariectomy

Prior to the experiments, the estrous cycle of the mice was synchronized by performing bilateral ovariectomy and provided with estrogen supplementation. This was carried out in 5-week-old mice under anesthesia induced by subcutaneous injection of a ketamine (100 mg/kg)/ xylazine-HCL (10 mg/kg) mixture in PBS (0.9%). Once the mice were anesthetized, a 1 cm incision was made unilaterally on the back beneath the costal margin. With a small opening in the peritoneal muscle, vessels were ligated, and both ovaries were resected. Then, a 4/0 braided nylon thread (Ethicon, Somerville, NJ, USA) was used to suture the incision site. Afterward, 100 ug/g of 17β-estradiol was subcutaneously (*s.c.*) injected into the ovariectomized mice. The mice were kept on warm bedding (37 °C) until recovery, after which they were returned to their cages. They were acclimatized for one week prior to transplantation surgery.

### 2.3. Experimental Endometriosis Model

Induction of endometriosis was performed according to our previous methods [23,24]. In brief, the endometrium was isolated from donor mice during the proliferating estrous stage, and endometrium biopsies 3 mm in diameter were obtained with a sterile dermal biopsy punch (Miltex, Lake Success, NY, USA). The biopsies were immediately washed in prewarmed, phenol-red-free DMEM/F-12 medium (Sigma Aldrich, Invitrogen Gibco, Carlsbad, CA, USA) to remove residual blood and mucus and were maintained in serum-free DMEM/F-12 medium before transplantation [25]. The endometrial biopsies were randomly allocated for transplantation, so three biopsies per treatment group were implanted into a single subcutaneous pocket created in the abdominal wall of the various recipient mice along the ventral midline immediately below the umbilicus. All the surgical procedures were performed under general anesthesia with isoflurane (Baxter, Deerfield, IL, USA), and the mice were monitored during and after transplantation until they fully recovered.

### 2.4. Macrophage Depletion

To verify the role of macrophages in angiogenesis and growth of endometriotic lesions, the systemic macrophage depletion was achieved by treatment with the anti-F4/80 antibody. For this purpose, the F4/80 Ab (0.8 μg/g/mice, Serotec, Kidlington, Oxfordshire, UK) antibody was intraperitoneally injected into female recipient C57Bl/6J mice two days before the transplantation [26]. Then, an additional injection was performed on the day of transplantation, and this procedure was repeated every other day for 3 weeks after transplantation. Mice injected with saline served as vehicle control.

### 2.5. Alox15 Knockout Mice

Alox15 is expressed at high levels in macrophages [27], and this pro-oxidant enzyme contributes to the redox equilibrium of the macrophages. To study whether oxidative stress-mediated macrophages might play a role in the angiogenesis and growth of endometriotic lesions, female Alox15^-/-^ mice [27] were used as recipient mice, and female wild-type C57BL/6J mice were used as donor mice. To synchronize the genetic background, homozygous Alox15^-/-^ mice were back-crossed (at least 8 times) with wild-type C57BL/6J mice [22]. C57BL/6J mice, wild-type recipient mice with the endometrium transplantation from another wild-type donor, served as control.

### 2.6. Hypoxia Inducible Factor-1alpha (HIF-1α) Inhibition

To further confirm the potential role of oxidative stress in endometrial angiogenesis, female C57Bl/6J recipient mice were treated with *i.p.* PX-468, a systemic anti-HIF-1α agent, (20 mg/kg/mice, Oncothyreon, Bellevue, Kentucky, USA), against hypoxia-induced oxidative stress two days before transplantation. On the day of transplantation, the mice received another dose of PX-468, and this treatment was repeated every other day for 3 weeks after transplantation. Mice injected with saline served as vehicle controls.

### 2.7. In Vivo Bioluminescence Imaging

Angiogenesis and development of the experimental endometriotic lesions were monitored using various in vivo imaging techniques. Growth of the endometriotic lesions was quantified at different timepoints by measuring the bioluminescence of subcutaneous UbC-Luc^+/+^ implants using a non-invasive IVIS 200 live animal imaging system (Xenogen, Baltimore, MD, USA) [25]. A total of 150 mg/kg of luciferin was intravenously injected into the mouse tail vein 10 min prior to imaging. IVIS imaging was carried out under general anesthesia with isoflurane (Baxter, IL, USA). An image of each animal was taken over a period of 120 s at a bin size of 4, and measurements were taken in triplicate. To quantify bioluminescence, identical circular regions of interest were positioned to encircle each luminescent lesion, and the integrated flux of photons (photons per second) within each region of interest was determined using the Living Images software package (Living Image 2.5, Xenogen, Baltimore, MD, USA) [25]. Data was normalized to the bioluminescence of each individual measured at the beginning of intervention.

In vivo oxidative stress and ROS formation were quantified employing the luminescent probe, L-012, 8-amino-5-chloro-7-phenyl-pyrido[3,4-d]pyridazine-1,4(2H,3H)dione (L-012), an analogue of luminol, as an ROS/RNS-sensor [28]. The luminescent probe was purchased from Wako Chemical (Neuss, Germany) and dissolved in ultrapure water. L-012 was subcutaneously administered at a dose of 25 mg/kg body weight (100 uL injection volume). Mice were kept anesthetized and warm in the IVIS200 chamber for 5 min. After 5 min, mice were imaged using the IVIS200 (Xenogen, Baltimore, MD, USA) for 2 s at bin size 4. The intensity of the luminescent signal was detected at different timepoints using an in vivo imaging system. All surgical procedures and imaging were performed under inhalation anesthesia with isoflurane. To quantify bioluminescence, identical circular regions of interest were positioned to the abdominal area of interest where the lesions were implanted, and the relative intensity area of photos within each region of interest was determined by using Living Image software (Living Image 2.5, Xenogen, Baltimore, MD, USA).

### 2.8. In Vivo Fibered Confocal Fluorescence Microscopic Imaging

In vivo visualization of the vasculature of the endometriotic lesions was performed using a minimally invasive fibered confocal fluorescence microscopy (FCFM) imaging system (Cellvizio^®^, Mauna Kea Technologies, Paris, France) using a Cellvizio LAB LSU-488 system with a ProFlex MiniO/100 (Mauna Kea Technologies, Paris) at different timepoints [25]. FITC-Dextran (MW 2000 kDa, 500 mg/kg, 150 μL) was intravenously injected immediately prior to FCFM imaging to visualize the microvascular network of the lesion surface. During the time of acquisition, mice were anesthetized with 1.5% isoflurane/98.5% oxygen and placed in the supine position. Body temperature was controlled by a heating mat. Images and videos were acquired using a ProFlex MiniO/100 microprobe above the endometriotic lesion. All imaging was carried out using a frame rate of 9 Hz (full FOV), a field of view of 618 × 609 μm, and 100% laser power at 488 nm. Images and videos were analyzed using Cellvizio^®^ dual viewer (Mauna Kea Technologies, Paris, France).

### 2.9. Histological Analysis and Immunohistochemistry

At the end of the experiments at different timepoints, the endometriotic lesions were prepared, fixed in 10% (*w*/*v*) phosphate-buffered formalin, and embedded in paraffin. Sections (4 µm thick) were cut and stained with hematoxylin and eosin to explore the histological structures. For immunohistochemistry, antigen was retrieved, and the slides were blocked with 3% hydrogen peroxide and 5% bovine serum albumin. The tissue sections were incubated overnight at 4 °C with the primary antibody diluted with PBS at 20, and a secondary antibody kit was utilized to link the primary antibody. A diaminobenzidine (DAB) staining kit (Dako, Copenhagen, Denmark) was used to develop positive staining. Details of the primary antibodies used are shown in Supplementary Table S1. Negative controls were conducted without using primary antibody.

### 2.10. Apoptosis in Endometriotic Lesions

For the quantitative analyses of apoptotic cell death, sections of paraffin-embedded lesions were assayed using the terminal deoxynucleotidyl transferase mediated dUTP-FITC nick end-labeling (TUNEL) method in conjunction with an apoptosis in situ detection kit (ApopTag^®^, Millipore, Wembley, Middlesex, UK) according to the manufacturer’s protocol. TUNEL-positive cells (mainly regarded as apoptotic cells) were counted in viable regions or peripheral to areas of necrosis in the early endometriotic lesion sections. The slides were scanned at low power (100×) magnification to identify areas having the highest number of TUNEL-positive cells. Four areas neighboring the highest area of TUNEL-positive cells were then selected and counted at higher (200× to 400×) magnification. The numbers of TUNEL-positive cells were expressed as numbers per cm^2^.

### 2.11. Detection of 8-Isoprostanes

The endometriotic lesions prepared at different timepoints were weighed and homogenized in 0.1 M phosphate buffer, containing 1 mM EDTA and 10 M indomethacin (pH 7.4), on ice. The supernatant was assayed by an ELISA method using an 8-isoprostane ELISA Kit (Cayman Chemicals, Ann Arbor, MI, USA), according to the manufacturer’s instructions. The numeric 8-isoprostane values were normalized to the protein content of the tissue homogenate supernatant, which was quantified by DC protein assay. The data obtained were then expressed as 8-isoptostane (in pg) per mg homogenate supernatant protein (in mg).

### 2.12. RNA Extraction and Quantitative RT-PCR

Total RNA was extracted from endometriotic tissues collected at different timepoints by homogenization of the tissue in TRI Reagent solution (Life Technologies, Carlsbad, CA, USA). After extraction, total RNA was purified using the RNeasy mini-column kit (Qiagen, Hilden, Germany). Complementary DNA was synthesized using the PrimeScript RT reagent kit (TaKaRa Bio, Otsu, Japan) with a template concentration of 100 ng total RNA. Real-time PCR was performed in a 10 μL total volume of reaction mixture containing 4 μL of cDNA, 5 μL of TaqMan Universal PCR Master Mix (Applied Biosystems, Foster City, CA, USA), 0.5 μL of TaqMan probes, and 0.5 μL of RNase-free H2O. The amplification conditions were as follows: 50 °C for 2 min, 95 °C for 15 min, followed by 45 cycles at 95 °C for 15 s and 60 °C for 60 s. Quantitative RT-PCR was performed using a 9700 real-time PCR machine (Applied Biosystems, Foster City, CA, USA), with GAPDH as the endogenous control. All reactions were carried out in triplicate. Negative controls were carried out by running samples involving all reagents but without the addition of a cDNA template. Primer sequences of the pre-designed TaqMan probes are shown in Supplementary Table S2.

### 2.13. Data Analysis

All data were expressed as relative mean ± standard error. Relative fold change was calculated by dividing the experimental value by the control. The sample size of each assay at each timepoint was performed in either quadruplicate or quintuplicate. Statistical significance of the difference between the control mice was analyzed with a parametric two-tailed Student’s t-test. Statistical significance of the difference from the baseline on the longitudinal changes in the same mouse on the same assay was determined by one-way analysis of variance (ANOVA) with post hoc LSD tests. The alpha value was set to 0.05 to be considered statistically significant. SPSS software (version 23; SPSS, Inc., Chicago, IL, USA) was used for statistical analysis.

## 3. Results

### 3.1. Lesional Angiogenesis in the Endometriosis Model

**Figure 1:**
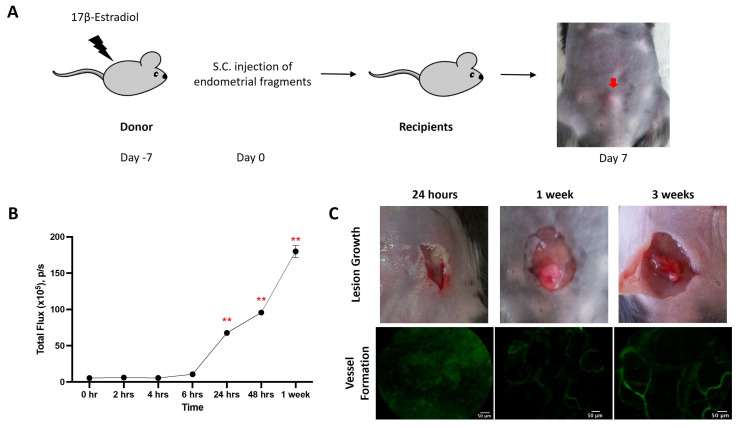
Mouse model of endometriosis. (**A**) Estradiol benzoate-treated female mice were sacrificed, and uteri were removed and split. Endometrial tissue was isolated and mechanically disrupted before subcutaneous injection. (**B**) The increase in cell density (measured by total flux) was evaluated by IVIS at different time points. (**C**) The increase in lesion size and vessel formation was evaluated by a caliper and Cellvizio, respectively, at different time points. Significantly different from baseline, ** *p* < 0.01.

Female C57BL/6 mice were implanted with endometriotic lesions from C57BL/6-UbC-Luc^+/+^ transgenic mice (Figure 1A). Luminescence signals were observed as early as 6 h after implantation, indicating the early development of vessels in the lesion (Figure 1B). The luminescence signals continued to rise and reached significance at 24 h, 48 h, and 1 week after transplantation. The lesions were attached between the outer abdominal layer and subcutaneous layer. In addition to the luminescence signals, vessel formation was verified by an in vivo confocal microscopy system (Cellvizio) in the corresponding time frame (Figure 1C).

### 3.2. Transient ROS Production in Early Development of Endometriosis

**Figure 2:**
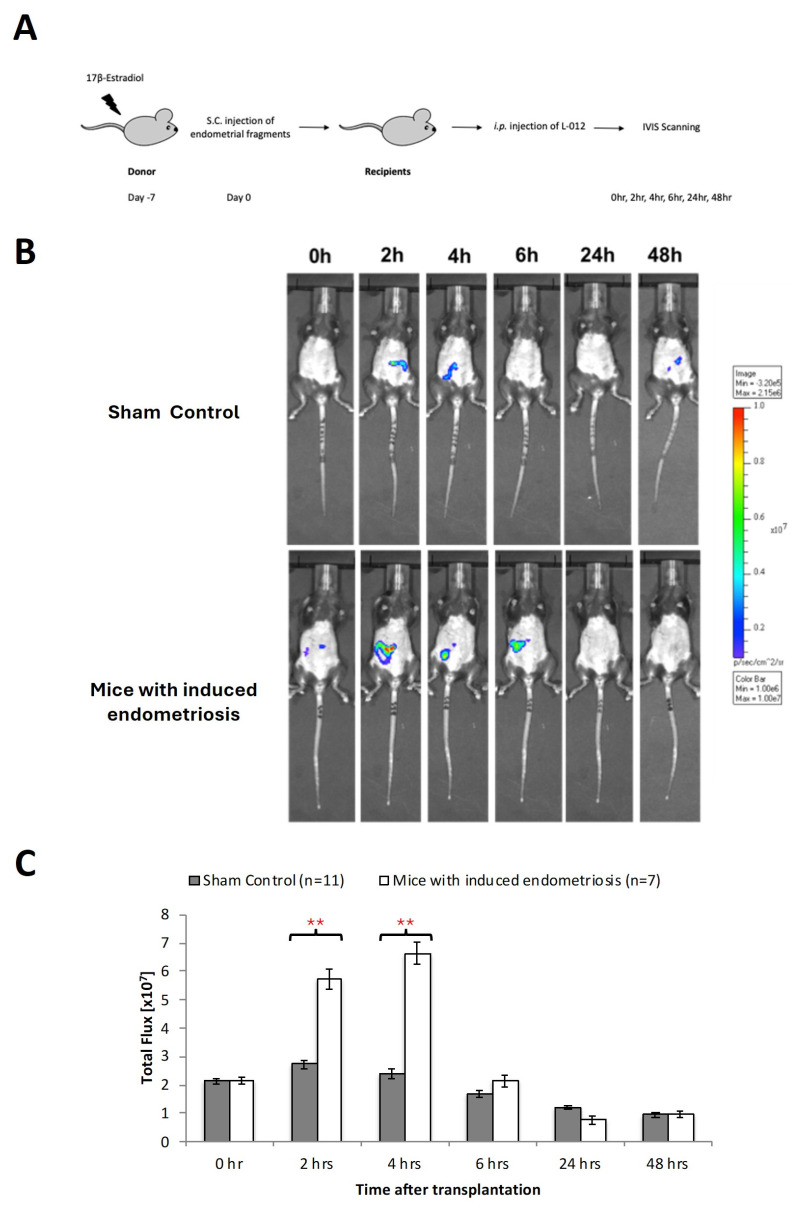
Oxidative stress in early endometriosis. (**A**) Estradiol benzoate-treated luciferase positive female mice were sacrificed and uteri removed and split. Endometrial tissue was isolated and mechanically disrupted before subcutaneous injection to a wild-type mouse. Wild-type mice without endometrial tissue injection were served as sham control. (**B**) Measurement of L-012 (ROS/RNS-sensing probe) showed peak intensity of ROS activity from 2 to 6 h. (**C**) Significant increase in oxidative stress detected by 8-isoprostane was found at 2 to 4 h between those with and without endometriotic bearing mice. Significantly different from control, * *p* < 0.05 and ** *p* < 0.01.

Using an imaging detection system, in vivo imaging of ROS formation was quantified at different timepoints after implantation of endometrial fragments followed by intravenous administration of L-012 (Figure 2A). Although we occasionally detected non-specific low-intensity signals in the sham-operated animals, these signals were mainly located in non-implanted areas (Figure 2B,C). In contrast, mice with endometriotic lesions showed strong ROS signals in the time interval between 2 and 4 h after implantation, and there were significant (*p* < 0.01) differences in the sham-operated individuals (Figure 2B,C). At later timepoints, the ROS signals began to decrease and reached initial values after 6 h (Figure 2C).

### 3.3. Transient Infiltration of Macrophages into the Ectopic Endometriosis Lesions

**Figure 3:**
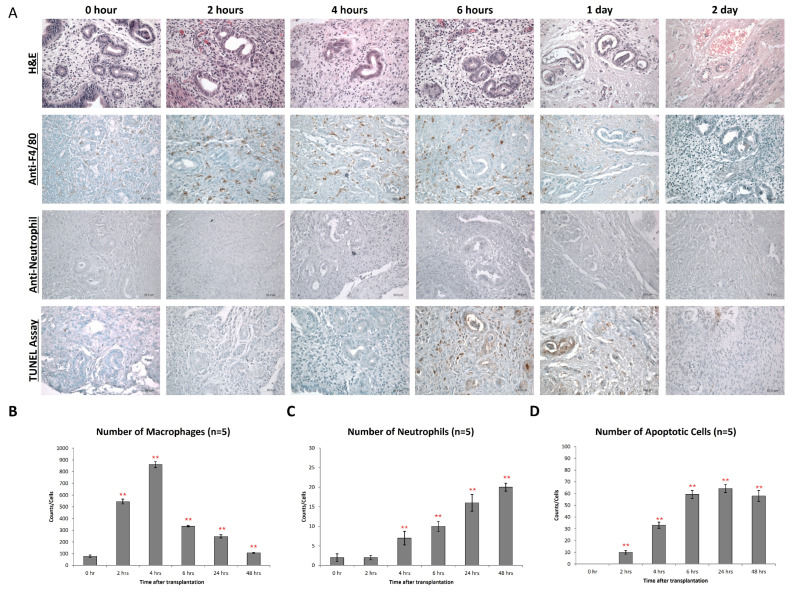
Macrophage infiltration during early endometriosis development. (**A**) Endometrial lesions were characterized by immunohistochemistry for the expression of F4/80 and neutrophil and presence of apoptosis. (**B**) Counts of F4/80+ cells (macrophages) in the endometrial tissue after transplantation at different time points. (**C**) Counts of neutrophils in the endometrial tissue after transplantation at different time points. (**D**) Counts of apoptotic cells in the endometrial tissue after transplantation at different time points. Significantly different from baseline, ** *p* < 0.01.

In parallel to the formation of ROS, we observed extensive macrophage infiltration into the endometriotic lesions (Figure 3). As ROS formation, macrophage infiltration peaked at 4 h (Figure 3A,B) but then gradually declined, reaching original macrophage counts at 48 h after implantation of the xenografts (Figure 3B). In contrast, the neutrophil count constantly increased during the time course of the experiments (Figure 3C), reaching a maximum of 2 days after implantation of endometriotic fragments. Apoptotic bodies were also found in both endometrial stroma and glandular epithelium from 4 h, and these structures remained until 2 days after transplantation (Figure 3D). At 24 h after implantation, we observed extensive necrosis, and glandular cells disappeared.

### 3.4. Macrophage Depletion Attenuated Early Endometriotic Angiogenesis and Lesion Development

**Figure 4:**
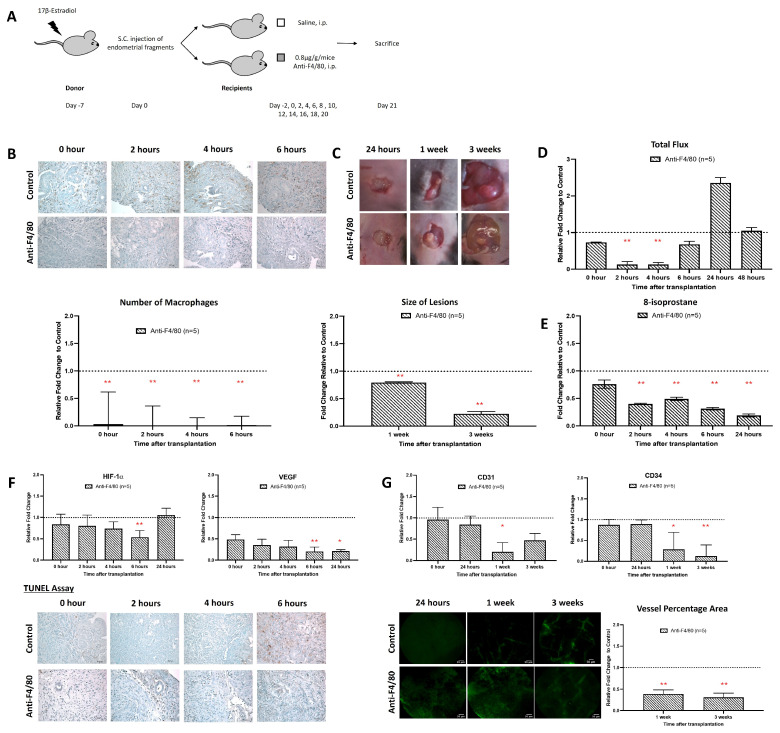
Inhibition of macrophages prevented the establishment of endometriosis. (**A**) Endometrial tissue was isolated and mechanically disrupted before subcutaneous injection of an experimental/control mice pair. Control mice and depleted mice were treated *i.p.* with PBS or anti-F4/80 24 h before. (**B**) Significantly lower relative number of macrophage infiltration in mice treated with anti-F4/80 than in the control. (**C**) The size and dry weight of endometriotic lesions in mice treated with anti-F4/80 were significantly lower than in the control at 1 and 3 weeks. (**D**) Measurement of L-012 showed relative peak intensity of ROS activity from 2 to 4 h in mice treated with anti-F4/80 than in the control. (**E**) Significantly lowered relative 8-isoprostane concentration in mice treated with anti-F4/80 than control at 2 to 24 h after transplantation. (**F**) Lower relative expression of HIF-1α and VEGF and lower apoptotic activity at 6 h in mice treated with anti-F4/80 than in the control. (**G**) Significantly lower vessel formation in mice treated with anti-F4/80 than in the control after 1 week and 3 weeks of transplantation. Relative fold change was calculated by dividing the experimental value by the control values. Significantly different relative to control, * *p* < 0.05 and ** *p* < 0.01.

The similar time course of macrophage infiltration and ROS formation suggested that macrophages might be the major source of ROS. To verify this assumption, we employed a macrophage-depletion strategy. For this purpose, recipient mice were repeatedly treated (*i.p.* injection) with an anti-macrophage antibody (anti-F4/80), which induces macrophage deficiency. As shown, it was found that anti-F4/80 treatment strongly reduced the number of macrophages in the endometriotic lesions (Figure 4B). Moreover, lesion formation was relatively significantly reduced in macrophage-depleted animals (Figure 4C). Most interestingly, we observed a strong relative reduction in ROS formation in the macrophage-depleted recipient mice (Figure 4D). A similar reduction was observed when we quantified the 8-isoprostane content as a suitable readout parameter. For oxidative stress, there were significant differences (*p* < 0.05) in the relative tissue concentration of 8-isoprostanes when normal recipient mice were compared with macrophage-depleted animals over the entire period of the experiment (Figure 4E).

During the early stage of endometriosis, hypoxia develops in the core of the endometriotic lesions, and such hypoxia usually triggers neoangiogenesis to improve oxygen supply [29]. Here, we found that macrophage depletion significantly suppressed the expression of the hypoxia marker HIF-1α and the angiogenesis marker VEGF (Figure 4F). We also observed a significant reduction in apoptotic cells in the lesions of macrophage-depleted animals 4 and 6 h after implantation (Figure 4F). Moreover, the degree of neovascularization was strongly reduced in macrophage-depleted mice when compared with control recipients (Figure 4G).

### 3.5. Alox15 Deficiency Limits Macrophage ROS Production and Growth of Endometriotic Lesions

**Figure 5:**
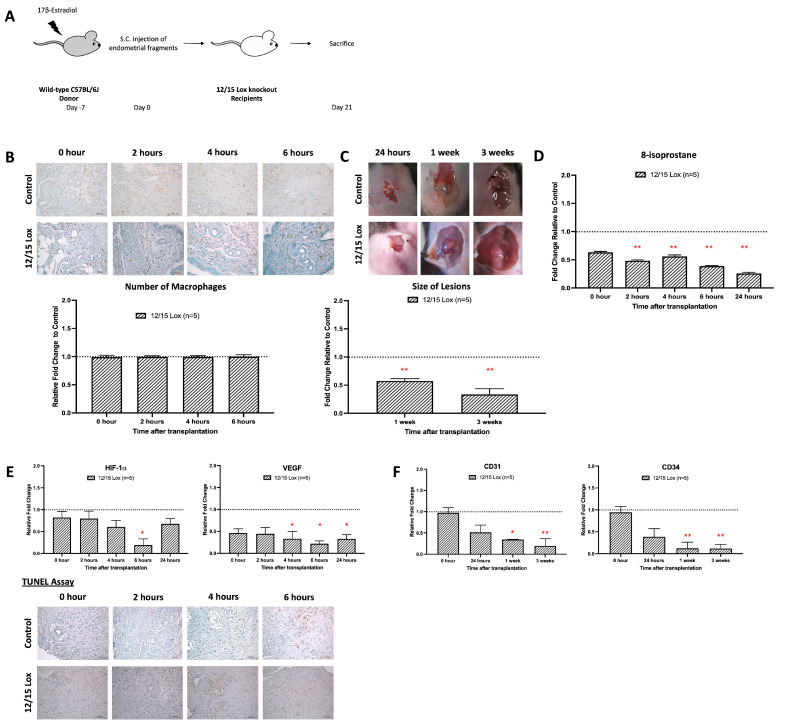
Absence of oxidative stress in the emerging role of the 12/15-Lipoxygenase knockout model inhibited the development of early endometrial development. (**A**) Endometrial tissue from wild-type mice was isolated and mechanically disrupted before subcutaneous injection of an experimental/control mice pair. Subcutaneous transplantation of endometrial fragments from wild-type mice to wild-type mice served as a control. (**B**) No observable difference in the relative number of macrophage infiltrations in mice treated with PX-478 and control. (**C**) The size and dry weight of endometriotic lesions in 12/15 Lox mice compared to the control was significantly lower at 1 and 3 weeks. (**D**) Significantly lowered relative 8-isoprostane concentration in 12/15 Lox mice than the control at 2 to 24 h after transplantation. (**E**) Lower relative expression of HIF-1α and VEGF and lower apoptotic activity at 6 h in 12/15 Lox mice than the control. (**F**) Significantly lower relative angiogenic expressions (CD31 and CD34) in 12/15 Lox mice than the control after 1 week of transplantation. Relative fold change was calculated by dividing the experimental value by the control value. Significantly different relative to control, * *p* < 0.05 and ** *p* < 0.01.

Alox15 is a pro-oxidant enzyme [30], which is at a high level expressed in mouse macrophages [27]. The enzyme oxidizes free and esterified polyenoic fatty acids to the corresponding hydroperoxides [31], and thus, it significantly contributes to the redox equilibrium of the macrophages. Disruption of the Alox15 pathway reduced oxidative modification of LDL in inflammatory processes [21,32]. Here, we employed Alox15^-/-^ mice to confirm whether functional inactivation of the macrophage-mediated oxidative stress might impact the angiogenesis of early endometriosis. Based on our model, the systemic inactivation of the Alox15 gene did not impact the degree of macrophage infiltration (Figure 5B). In contrast, the size of the endometriotic lesions was relatively significantly reduced in the Alox15^-/-^ (Figure 5C), and the 8-isoprostane levels were also attenuated (Figure 5D). In appearance, the lesions looked less vascularized (Figure 5C), and this observation was supported by the strongly reduced relative expression of the neovascularization marker VEGF (Figure 5E). These data suggest that neoangiogenesis is impaired in Alox15^-/-^ mice, which might be a reason for reduced lesion growth and development (Figure 5C).

We also observed that expression of the hypoxia marker HIF-1α was reduced in Alox15^-/-^ mice at each timepoint of the experimental protocol, but only on day 6 was this difference statistically significant (Figure 5E, left panel). Finally, we quantified the degree of apoptosis (TUNEL assay) in the endometriotic lesions 4 and 6 h after implantation and found fewer apoptotic cells in the lesional areas of Alox15^-/-^ mice. When we compared the degree of neovascularization of wild-type mice with that of Alox15^-/-^ animals (Figure 5F), we detected a normal degree of vessel formation in the control animals, which is clearly indicated by the relative expression of the neovascularization markers CD31 and CD34. Interestingly, neovascularization was completely inhibited in Alox15^-/-^ mice over the entire period of the experiment.

### 3.6. HIF-1α Inhibition Interferes with Early Development and Vascularization in Endometriosis

**Figure 6:**
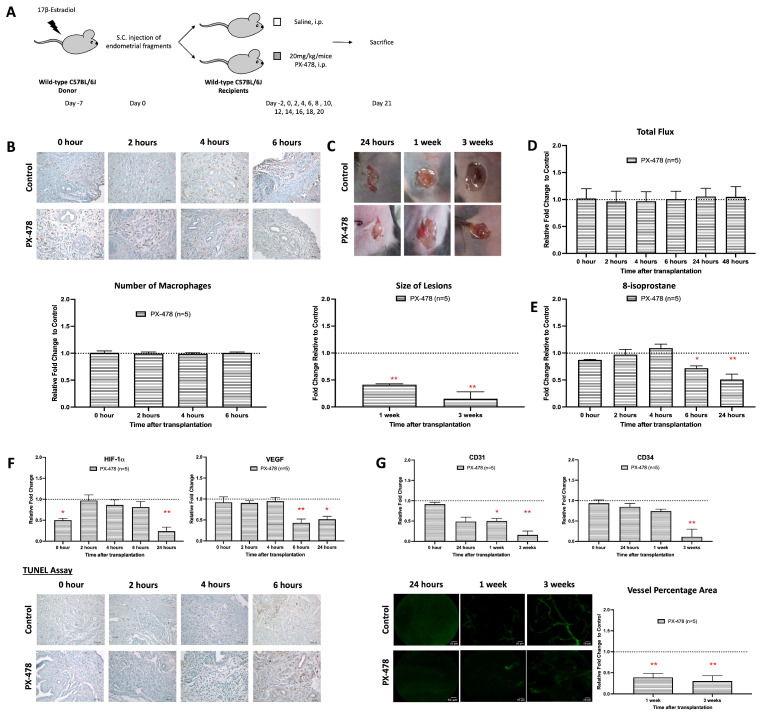
HIF-1 inhibitor suppresses growth and angiogenesis during the development of endometriosis. (**A**) Endometrial tissue was isolated and mechanically disrupted before subcutaneous injection of an experimental/control mice pair. Control mice and depleted mice were treated *i.p.* with PBS or PX-478 (HIF-1 inhibitor) 24 h before. (**B**) The size and dry weight of endometriotic lesions in mice treated with PX-478 were significantly lower than the control at 1 and 3 weeks. (**C**) Measurements of relative L-012 were similar between mice treated with PX-478 and control. (**D**) Significantly lowered 8-isoprostane relative concentration in mice treated with PX-478 compared to the control at 6 to 24 h after transplantation. (**E**) No observable difference in the number of macrophage infiltrations in mice treated with PX-478 and the control. (**F**) Lower relative expression of HIF-1α and VEGF and higher apoptotic activity in mice treated with PX-478 than in the control. (**G**) Significantly lower vessel formation in mice treated with anti-F4/80 than in the control after 3 weeks of transplantation. Relative fold change was calculated by dividing experimental values with control values. Significantly different relative to control, * *p* < 0.05 and ** *p* < 0.01.

HIF-1α is a transcription factor that regulates the expression of many proteins that are involved in the adaptation of cellular mechanisms under hypoxic conditions [33]. In macrophages, it also alters the redox equilibrium via upregulation of the formation of ROS [34]. To investigate whether inhibition of the HIF-1α activity impacts the angiogenesis of early endometriosis, mice carrying endometriotic lesions were treated with a HIF-1 inhibitor (PX-478), and the extent of lesion formation and angiogenesis was quantified (Figure 6A). Like the anti-F4/80 treatment and Alox15 deficiency, the PX-478 treatment significantly inhibited lesion formation (Figure 6B). In contrast, PX-478 neither modified the relative formation of ROS (Figure 6C) nor the tissue relative levels of 8-isoprostanes during the early stage of lesion development (Figure 6D). Thus, in this experimental model, inhibition of HIF-1α does not modify the redox equilibrium during the early stages of lesion development. However, at later stages (6 h and 24 h), higher relative 8-isoprostane levels were detected in mice treated with PX-478 (Figure 6D). In addition, inhibition of HIF-1α did not alter the extent of macrophage infiltration into the endometriotic lesions (Figure 6E), and during early stages of lesion formation we did not observe any alterations in the expression of HIF-1α and VEGF (Figure 6F). However, at later stages of lesion development (6 h and 24 h), HIF-1α and VEGF-A expressions were significantly reduced by PX 478 treatment (Figure 6F). Finally, we quantified the impact of PX-478 treatment on the degree of neovascularization (Figure 6G). Here, we observed a completely prevented neovascularization in the PX-478-treated mice, which was indicated by downregulated expressions of CD31 and CD34 with a reduced percentage of neovascularized lesional area.

## 4. Discussion

Endometriosis is a frequent female disorder, and because of its abundance, it is of major socio-economic impact. Unfortunately, our knowledge of the pathophysiological mechanisms of this disease is rather limited, and for the time being, there is only symptomatic therapy. Here, we explore the possible relevance of macrophage-induced oxidative stress on the development and maintenance of subcutaneous endometriotic lesions. We found that macrophage depletion (Figure 4), Alox15 knockout (Figure 5), and HIF-1α inhibition (Figure 6) strongly impaired the development of endometriotic lesions, and this observation might be related to the reduced degree of neovascularization and to an increased extent of cellular apoptosis. According to our data, macrophages and their oxidative metabolism may play a major role in the pathogenesis of this disease, and thus, targeted interference of macrophage functionality might constitute an innovative principle for treating endometriosis. However, considering the important immune functions of macrophages, depletion of these cells is likely to cause severe adverse effects. Moreover, since cellular redox homeostasis is important for expression of redox-sensitive genes, pharmacological interference with the cellular redox state might also induce adverse side effects, and thus, this line of intervention might not be suitable. PX-478 is a small-molecule inhibitor of HIF-1α that has demonstrated antitumor activity in subcutaneous xenograft models [35]. However, no previous studies have demonstrated the activity of this compound in endometriosis. We clearly showed the effectiveness of the HIF-1α inhibitor (PX-478) in deferring lesion growth in our endometriotic mouse model with no observable adverse side effects.

Alox15 is a pro-oxidant enzyme that is constitutively expressed at high levels in mouse peritoneal macrophages [27]. When activated, it oxidizes free and esterified polyunsaturated fatty acids to corresponding hydroperoxy lipids [30]. Under normal conditions, these hydroperoxyl lipids are rapidly reduced to less harmful hydroxy compounds preferentially by glutathione peroxidase 4 (Gpx4), and thus, Gpx4 is an effective regulator of the Alox15 pathway [36]. However, when the reduced capacity of the cells is overcome by oxidative challenge, Alox15-derived hydroperoxyl lipids may accumulate and undergo secondary decomposition reactions that involve the formation of ROS. Here, we show for the first time that systemic functional inactivation of the Alox15 gene inhibited the formation of endometriotic lesions (Figure 5C) and that this effect might be related to almost complete prevention of neovascularization and strongly induced apoptosis (Figure 5E,F). These data suggest that pharmacological interference with the Alox15 pathway might constitute an innovative approach for the treatment of endometriosis. Unfortunately, for the time being, isoform-specific ALOX inhibitors are currently not available [37]. Moreover, since mouse Alox15 exhibits different catalytic properties than its human ortholog [38] and since the sensitivity of the two enzymes for ALOX inhibitors is quite different [37], it remains unclear whether the results obtained in our mouse experimental model system can directly be transferred to the human situation.

The HIF-1 transcription factor is an important regulator of the response of endometriotic lesions toward hypoxia, and this response involves increased angiogenesis, glycolytic metabolism, and resistance to apoptosis. The transcription factor activity of HIF1 is regulated by the availability of the HIF-1α subunit, the levels of which increase under hypoxic conditions. Hence, if one aims at reducing the growth and development of endometriotic lesions, it would be a suitable strategy to target HIF signaling. In previous studies, it has been shown that the HIF1 inhibitor PX-478 exhibits antitumor activity [35,39]. Here, we show for the first time that xenografts treated with PX-478 develop significantly smaller lesions when compared with corresponding control xenografts, which were not treated with PX-478.

During angiogenesis, the hypoxic state of the endometrium contributes to the development of endometriosis. In our mouse model, we showed that oxidative stress was increased in endometriotic lesions during the early stages of transplantation. The production of ROS was increased as early as 2 to 4 h after transplantation. At the same time, expression of HIF-1α was also increased. This was followed by an increase in 8-isoprostane later, 1 day after transplantation. These data suggest that ROS rapidly induces HIF-1α expression and subsequently activation of the angiogenic factor, VEGF. Hence, oxidative stress is an upstream event in the signaling cascade responsible for neovascularization of endometriotic lesions, and this conclusion supports previous experimental findings [40]. Likewise, antiangiogenic agents 2-methylestradiol (a natural metabolite of estradiol) and 2-hydroxyestradiol (2-OHE2) inhibited HIF-1α and decreased the lesion size [40].

As shown in previous literature, ROS and HIF-1α are interconnected in their roles during hypoxia [41]. ROS are produced by the mitochondrial electron transport chain and are necessary for critical period-regulated plasticity [42]. Under hypoxic conditions, HIF-1α induces adaptive changes in cell metabolism, including the activation of glycolysis pathways. Evidence suggests the ROS produced particularly by mitochondrial complex III is responsible for stabilizing HIF-1a during hypoxia. Suppressing the Rieske iron–sulfur protein of complex III in HEK293 and Hep3B cells increased both ROS production and HIF-1a stabilization under hypoxia [43,44]. This signaling axis is crucial for cell behavior and tissue integrity, highlighting the concurrent role of hypoxia in these processes, which underscores the importance of ROS and HIF-1α in mediating cellular responses to hypoxia.

Macrophages have been widely shown to play a key role in regulating immune response. Studies addressed the role of inflammatory factors, especially the role of macrophages, in the adhesion and growth of endometrial tissue [45]. Clinical studies reported increases in macrophages and inflammatory cytokines in the peritoneal fluid of women with endometriosis [9,46]. The specificity of macrophage depletion with anti-F4/80 in the endometriosis mice model has been confirmed through various studies [47]. The F4/80 marker is a cell surface glycoprotein that is highly expressed on mature tissue macrophages, including those in the peritoneal cavity, gut, kidney, and lymph nodes. Its expression can vary depending on the type of macrophage and its state of maturation or activation. In the context of endometriosis, F4/80-positive macrophages are known to play a significant role in immune response and tissue repair. A study has shown that the depletion of these macrophages using anti-F4/80 antibodies can lead to smaller endometriosis lesions, while constitutive inhibition of monocyte recruitment significantly reduces peritoneal macrophage populations and increases the number of lesions. This suggests that macrophages play a dual role in endometriosis, with pro-endometriosis functions from the eutopic endometrial macrophages and anti-endometriosis functions from monocyte-derived macrophages. Similarly, our data also suggests that the anti-F4/80 antibodies are effective in identifying and depleting the specific macrophage subsets that are critical for early endometriosis development and progression (Figure 4).

To understand the underlying mechanism of oxidative stress mediated by macrophages during the development of endometriosis, Alox15^-/-^ mice were used. Lipoxygenases (LOX) constitute a family of lipid-peroxidizing enzymes that catalyze the oxygenation of polyunsaturated fatty acids to their corresponding hydroperoxyl derivatives [48]. Alox15^-/-^ mice do not show obvious abnormalities in reticulocytes or mature red blood cells but specifically abolished oxidation activity of phospholipids and lipoproteins in macrophages [21]. There is a functional difference between mouse Alox15 and human *ALOX15* that lies in their enzymatic properties and biosynthetic capacities. In mice, Alox15 forms 15-hydroperoxy arachidonic acid, which may exhibit improved biosynthetic capacity for pro-resolving mediators compared to human *ALOX15* [49]. While in humans, *ALOX15* is characterized by its role in generating specific phospholipid oxidation products crucial for nonimmunogenic cell removal and the production of specialized pro-resolving mediators [49]. The catalytic properties of *ALOX15* isoforms differ, with human ALOX15 being more effective in synthesizing pro-resolving mediators, while mouse Alox15 may have different substrate preferences. These differences highlight the importance of ALOX15 in inflammation resolution and immune response, with implications for understanding inflammatory diseases. Although the general function of Alox15^-/-^ macrophages was hardly impaired, the cells were defective in ROS formation, and these conclusions were consistent with the reduced 8-isoprostane formation observed in the endometriotic lesions (Figure 5). In endometriotic lesions of Alox15^-/-^ mice, oxidative stress is reduced, activation of HIF-1 is prevented, and neoangiogenesis is attenuated.

**Figure 7:**
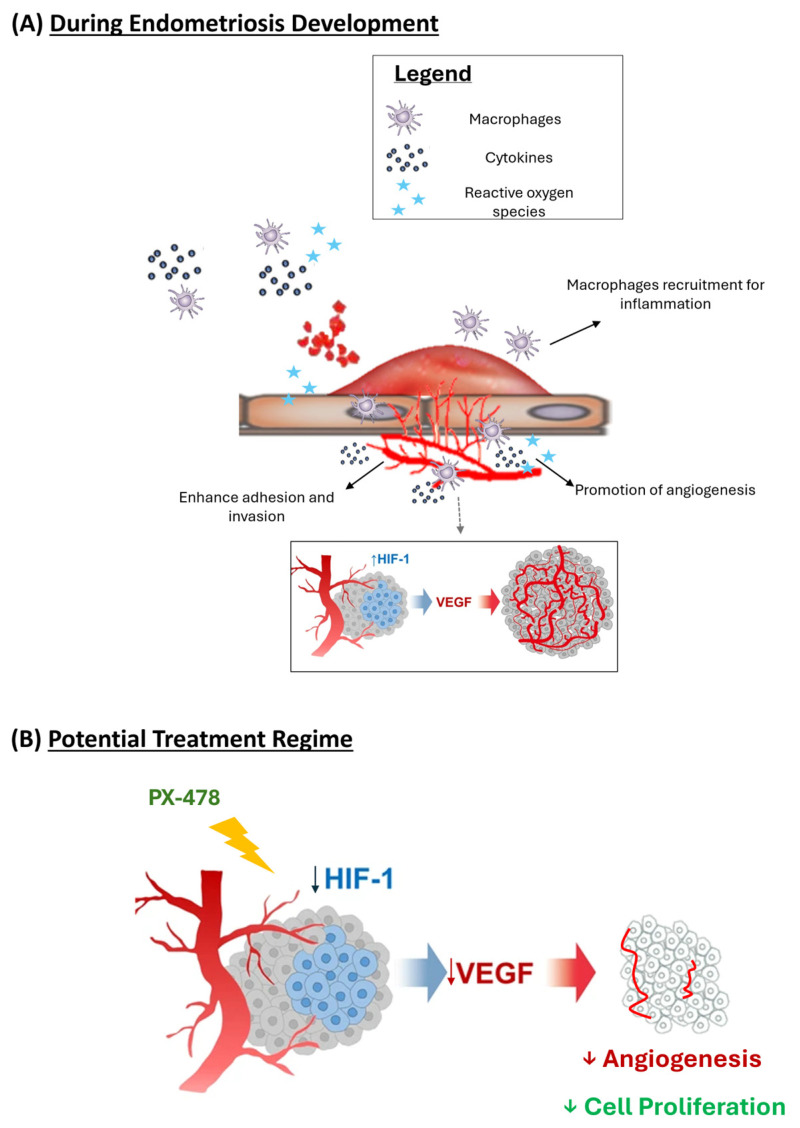
**Schematic diagram on the formation of endometriosis**. (**A**) Early growth and vascular development of endometriosis. (**B**) Proposed mechanism on PX-478 on preventing early endometriosis formation.

The importance of macrophages and oxidative stress in promoting lesion development and angiogenesis during endometriosis provides potential for endometriosis treatment in humans. Inhibition of macrophages would not be possible clinically, as this might impair the functionality of the immune system. However, more downstream inhibition of the Alox15-oxidative stress–HIF–neoangiogenesis signaling cascade would be more appropriate. We have previously reported that PX-478 exhibited antiangiogenic and antitumor activity in a variety of human tumor xenografts [35,39], but the effect of this compound on spreading over endometriotic cells has never been explored before. Thus, this is the first study to use PX-478 as a therapeutic approach for endometriosis. We here describe those mice treated with PX-478 for 3 weeks who developed significantly smaller endometriotic lesions when compared with sham-treated animals. The decrease in the lesion growth was paralleled by an impaired production of VEGF, which plays an important role in endometriosis [24,40,50]. Similarly, we observed reduced expression levels of the angiogenic and blood vessel-forming markers CD31 and CD34. In fact, after 3 weeks of treatment, there was no CD31 expression anymore. These results indicated that the antiangiogenic properties of PX-478 could be potentially useful for the treatment of endometriosis (Figure 7).

To explore the mechanistic basis for implantation of endometriotic cells in extrauterine environments in humans and animals, an in vivo model is required. This model system should mirror the mechanistic events of human endometriosis as closely as possible. Thus, non-human primate models would be the first choice, but the use of such highly developed animals would raise ethical and economic concerns. On the other hand, rodent endometriosis models (rats, mice) suffer from the disadvantage that these animals do not have menstruation and never develop spontaneous endometriosis. Nevertheless, our model closely mimics the human condition since lesion growth was estrogen-dependent and, as in human endometriotic lesions, we observed a strong inflammatory reaction. Also, the experimental endometriosis model was established by subcutaneous rather than intraperitoneal transplantation. As most endometriosis occurs in the peritoneal cavity, an intraperitoneal approach can represent the growth and development of endometriosis as it occurs in endometriosis patients. As compared by our team, both the endometriosis mouse model by intraperitoneal transplantation and the model by subcutaneous transplantation show similar histological characteristics of human endometriosis, including the presence of glands and stroma [25,51]. The primary limitation of using a subcutaneous model rather than an intraperitoneal model in endometriosis research is the inability to model spontaneous metastasis, including local invasion, intravasation, circulation, extravasation, and colonization. This lack of metastasis limits the model’s ability to accurately reflect the immune response and the progression of endometriosis in the abdominal cavity. However, intraperitoneal transplantation results in random implantation sites, which increase the variation in bioluminescence images for accurate quantitation and for consistent comparisons within and among groups. Although endometrial tissues can be fixed in the subperitoneal wall by sutures, as previously described [40], the sutures themselves can further damage the tissues and affect the angiogenesis process. In addition, the established endometriotic lesions tend to be very small and embedded in the abdominal organs in the intraperitoneal transplantation model, making them difficult to retrieve, and it is not easy to monitor lesion growth without opening the abdomen. Therefore, a subcutaneous transplantation protocol and suture-free procedure are preferable. And as previously shown by our team, we have utilized this experimental in vivo model successfully for studying angiogenesis mechanisms and micro-vessel imaging in endometriosis [25]. Also, the early ROS signal detected by L-012 may indeed originate from non-macrophage sources, such as surgical trauma. Research indicates that L-012 can detect ROS generated by various biological systems, including neutrophils, which are crucial for inflammation and tissue repair. Yet, as we have utilized five samples and sham controls in the experiment, this can help to minimize the effect of early ROS signal resulting from the surgical implantation. Similarly, the use of other probes (e.g., NIR-ICG) can be implemented to contextualize the role of angiogenesis and vascular remodeling in the clinical visualization of endometriotic lesions [52]. In contrast, our findings showed that the 8-isoprostane levels were also attenuated in the Alox15^-/-^ at early timepoints (Figure 5). This resulted in the lesions being less vascularized (Figure 5C), suggesting that neoangiogenesis is impaired in Alox15^-/-^ mice, which might be a reason for reduced lesion growth and development. Nonetheless, further prospective studies across diverse inflammatory pathologies will be essential to define specificity and avoid false-positive interpretations.

Moreover, as in human endometriotic lesions, we detect massive macrophage infiltration in the mouse lesions. It should also be noted that within the peritoneal cavity of mice, there are distinct subclasses of peritoneal macrophages. Large peritoneal macrophages (LPMs) are F4/80^+^MHCII^-^ and can be either tissue resident or monocyte derived; however, small peritoneal macrophages (SPMs) are F4/80-/MHCII^+^. Therefore, the depletion of only F4/80^+^ macrophages would lead to only the depletion of LPMs. It is worth considering/discussing that only a certain type of macrophage could be depleted by this method and the possible consequences. Hence, the validation of the depletion of macrophages within the peritoneal cavity using flow cytometry of the peritoneal fluid of these mice would serve to assess the type of macrophage depleted (i.e., LPM and SPM). Although we showed that macrophages played an essential role in producing ROS to promote hypoxia in our experimental endometriosis model to initiate angiogenesis, the type of macrophage has not been determined. According to their functional state, macrophages are usually classified as M0 (naive macrophages), M1 (pro-inflammatory macrophages), or M2 (anti-inflammatory macrophages) [53,54]. Naive M0 macrophages develop into M1 macrophages when stimulated with classical pro-inflammatory cytokines (such as interleukin 1 or 6 (IL-1, IL-6)). In contrast, when stimulated with IL-4 or IL-13 M0 (alternative stimuli), macrophages develop into M2. M1 macrophages (classically activated macrophages) are parts of the acute innate immune response. They phagocytose pathogens and produce pro-inflammatory cytokines [53]. In contrast, M2 macrophages play an essential role in inflammatory resolution, in wound healing, and in tissue repair. They phagocytose apoptotic immune cells and clean up the inflammatory battlefield, providing an environment for tissue restructuring. In other words, they terminate the acute immune reaction by producing anti-inflammatory cytokines and initiate tissue repair [55]. M1 macrophages are present in large quantities in tissues during the acute phase of inflammation. In contrast, during inflammatory resolution, M2 macrophages prevail. In chronic inflammation, M1 and M2 macrophages coexist. In endometriosis, M2 macrophages are anti-inflammatory and immunoregulatory [56]. The aberrant increase and activation of the anti-inflammatory M2 macrophage stimulates the abnormal gene expression that is associated with ectopic endometrium [57]. Moreover, M2 macrophages are elevated in III-IV endometriosis [58]. The uncharacterized macrophage polarization (M1/M2) is a significant limitation in this study, which can lead to inaccurate conclusions and hinder the understanding of immune responses. Herein, further experiments are needed to explore whether M1 or M2 macrophages were responsible for the oxidative stress during early development of endometriosis. Such further studies should focus on investigating the role of Alox15 in M2 phenotypes to better understand its impact on macrophage polarization and its potential therapeutic targets, developing standardized methods for detecting and quantifying macrophage polarization to ensure consistency and reliability in studies, utilizing advanced technologies such as algorithms and machine learning to analyze macrophage polarization data more effectively, and exploring the link between macrophage polarization and various diseases, including autoimmune diseases, to identify new therapeutic strategies. By addressing these limitations and exploring the potential of Alox15, it may help to advance the understanding of macrophage polarization and its implications for health and disease.

Whereas for clinical translation, VEGF and its related pathway have served as primary targets for antiangiogenic therapy. However, the use of an anti-VEGF antibody or VEGFR inhibitors as antiangiogenic therapy was found not to be very optimistic, owing to effectiveness and safety [59]. Despite the great importance of VEGF during oxidative stress-induced angiogenesis, there are a variety of VEGF-independent signaling pathways implicated in pathological angiogenesis, such as the ROS/ATM/p38α and CEP/TLR2/MyD88 pathways, which may reduce the efficacy of anti-VEGF therapy [15]. On the other hand, inhibition of the VEGF pathway may disturb the homeostatic maintenance of normal vasculature and result in delayed wound healing, hypertension, proteinuria, thrombosis, and hemorrhage. One possibility is the use of a preclinical inhibitor of HIF-1α (PX-478), which is the main regulator for promoting neovascularization. However, the studies using such inhibitors in in vivo animal models are limited (with no clinical studies). Though it showed promising lesion suppression in our study, it remains uncertain whether the effects are nonspecific to affect both pathological and physiological angiogenesis. Hence, it is difficult to determine the optimal dose of the inhibitor for each pathological condition in each individual. Therefore, there is a need for a better understanding of the precise molecular mechanisms behind oxidative stress-induced angiogenesis and the identification of markers and processes specific to either pathological or physiological angiogenesis. In addition, prior to any clinical assessments, further developments to use an antiangiogenic drug in combination with PX-478 in the treatment of endometriosis for inhibiting vascularization and inducing hypoxia, which may further defer the growth of endometriosis lesions, are still warranted.

## 5. Conclusions

In this study, we have demonstrated that macrophages play a major role in the angiogenesis of endometriosis. Macrophage depletion, inactivation, and pharmacological inhibition of macrophage-mediated ROS signaling prevent the angiogenesis and development of endometriosis in the murine implantation models. Moreover, we showed that the preclinical HIF-1α inhibitor, PX-478, can effectively prevent endometriosis growth and development, with significant reduction in vascularization, indicating therapeutic potential for prevention and treatment of endometriosis in the future.

## Data Availability

All data generated or analyzed during this study are available from the corresponding author upon reasonable request.

## Supplementary Materials

The following supporting information can be downloaded at https://www.mdpi.com/article/10.3390/antiox15020159/s1, Supplementary Table S1. Primary antibodies for semi-quantitative analysis. Supplementary Table S2. TaqMan primer sequences used for quantitative real-time analysis.

## Abbreviations

The following abbreviations are used in this manuscript:

12/15-LOX	12/15-lipoxygenase
ANOVA	One-way analysis of variance
cDNA	Double-stranded complementary DNA
DAB	Diaminobenzidine
DC protein	Detergent-compatible protein
EDTA	Ethylenediaminetetraacetic acid
ELISA	Enzyme-linked immunosorbent assay
FCFM	Fibered confocal fluorescence microscopy
FITC	Fluorescein isothiocyanate
FOV	Field of view
GAPDH	Glyceraldehyde 3-phosphate dehydrogenase
H&E	Hematoxylin and eosin
HIF-1α	Hypoxic inducible factors-1 alpha
HRP	Horseradish peroxidase
*I.P.*	Intraperitoneal
LOX	Lipoxygenases
mRNA	Messenger RNA
MW	Molecular weight
NK cells	Natural killer cells
OVX	Ovariectomized
PBS	Phosphate-buffered saline
qPCR	Quantitative polymerase chain reaction
RNA	Ribonucleic acid
ROS	Reactive oxygen species
RT-PCR	Reverse transcriptase polymerase chain reaction
*S.C.*	Subcutaneous
SCID mice	Severe combined immunodeficient mice
Tregs	Regulatory T cells
TUNEL	Terminal deoxynucleotidyl transferase-mediated dUTP-FITC nick end-labeling
VEGF	Vascular endothelial growth factor

## References

[1] Taylor H.S., Kotlyar A.M., Flores V.A. (2021). Endometriosis is a chronic systemic disease: Clinical challenges and novel innovations. Lancet.

[2] Sampson J.A. (1927). Metastatic or Embolic Endometriosis, due to the Menstrual Dissemination of Endometrial Tissue into the Venous Circulation. Am. J. Pathol..

[3] Halme J., Hammond M.G., Hulka J.F., Raj S.G., Talbert L.M. (1984). Retrograde menstruation in healthy women and in patients with endometriosis. Obstet. Gynecol..

[4] Ahn S.H., Monsanto S.P., Miller C., Singh S.S., Thomas R., Tayade C. (2015). Pathophysiology and Immune Dysfunction in Endometriosis. BioMed Res. Int..

[5] Saunders P.T.K., Horne A.W. (2021). Endometriosis: Etiology, pathobiology, and therapeutic prospects. Cell.

[6] Zhang T., De Carolis C., Man G.C.W., Wang C.C. (2018). The link between immunity, autoimmunity and endometriosis: A literature update. Autoimmun. Rev..

[7] Chan R.W.S., Lee C.L., Ng E.H.Y., Yeung W.S.B. (2017). Co-culture with macrophages enhances the clonogenic and invasion activity of endometriotic stromal cells. Cell Prolif..

[8] Abramiuk M., Grywalska E., Malkowska P., Sierawska O., Hrynkiewicz R., Niedzwiedzka-Rystwej P. (2022). The Role of the Immune System in the Development of Endometriosis. Cells.

[9] Hogg C., Panir K., Dhami P., Rosser M., Mack M., Soong D., Pollard J.W., Jenkins S.J., Horne A.W., Greaves E. (2021). Macrophages inhibit and enhance endometriosis depending on their origin. Proc. Natl. Acad. Sci. USA.

[10] Johan M.Z., Ingman W.V., Robertson S.A., Hull M.L. (2019). Macrophages infiltrating endometriosis-like lesions exhibit progressive phenotype changes in a heterologous mouse model. J. Reprod. Immunol..

[11] Lebovic D.I., Mueller M.D., Taylor R.N. (2001). Immunobiology of endometriosis. Fertil. Steril..

[12] Harada T., Iwabe T., Terakawa N. (2001). Role of cytokines in endometriosis. Fertil. Steril..

[13] Chiu Y.C., Chu P.W., Lin H.C., Chen S.K. (2021). Accumulation of cholesterol suppresses oxidative phosphorylation and altered responses to inflammatory stimuli of macrophages. Biochem. Biophys. Rep..

[14] Moghaddam M.Z., Ansariniya H., Seifati S.M., Zare F., Fesahat F. (2022). Immunopathogenesis of endometriosis: An overview of the role of innate and adaptive immune cells and their mediators. Am. J. Reprod. Immunol..

[15] Kim Y.W., West X.Z., Byzova T.V. (2013). Inflammation and oxidative stress in angiogenesis and vascular disease. J. Mol. Med..

[16] Carvalho L.F., Samadder A.N., Agarwal A., Fernandes L.F., Abrao M.S. (2012). Oxidative stress biomarkers in patients with endometriosis: Systematic review. Arch. Gynecol. Obstet..

[17] Turkyilmaz E., Yildirim M., Cendek B.D., Baran P., Alisik M., Dalgaci F., Yavuz A.F. (2016). Evaluation of oxidative stress markers and intra-extracellular antioxidant activities in patients with endometriosis. Eur. J. Obstet. Gynecol. Reprod. Biol..

[18] Prieto L., Quesada J.F., Cambero O., Pacheco A., Pellicer A., Codoceo R., Garcia-Velasco J.A. (2012). Analysis of follicular fluid and serum markers of oxidative stress in women with infertility related to endometriosis. Fertil. Steril..

[19] Jackson L.W., Schisterman E.F., Dey-Rao R., Browne R., Armstrong D. (2005). Oxidative stress and endometriosis. Hum. Reprod..

[20] Pacher P., Beckman J.S., Liaudet L. (2007). Nitric oxide and peroxynitrite in health and disease. Physiol. Rev..

[21] Sun D., Funk C.D. (1996). Disruption of 12/15-lipoxygenase expression in peritoneal macrophages. Enhanced utilization of the 5-lipoxygenase pathway and diminished oxidation of low density lipoprotein. J. Biol. Chem..

[22] Rademacher M., Kuhn H., Borchert A. (2020). Systemic deficiency of mouse arachidonate 15-lipoxygenase induces defective erythropoiesis and transgenic expression of the human enzyme rescues this phenotype. FASEB J..

[23] Xu H., Zhang T., Man G.C., May K.E., Becker C.M., Davis T.N., Kung A.L., Birsner A.E., D’Amato R.J., Wong A.W. (2013). Vascular endothelial growth factor C is increased in endometrium and promotes endothelial functions, vascular permeability and angiogenesis and growth of endometriosis. Angiogenesis.

[24] Xu H., Lui W.T., Chu C.Y., Ng P.S., Wang C.C., Rogers M.S. (2009). Anti-angiogenic effects of green tea catechin on an experimental endometriosis mouse model. Hum. Reprod..

[25] Wang C.C., Xu H., Man G.C., Zhang T., Chu K.O., Chu C.Y., Cheng J.T., Li G., He Y.X., Qin L. (2013). Prodrug of green tea epigallocatechin-3-gallate (Pro-EGCG) as a potent anti-angiogenesis agent for endometriosis in mice. Angiogenesis.

[26] Bacci M., Capobianco A., Monno A., Cottone L., Di Puppo F., Camisa B., Mariani M., Brignole C., Ponzoni M., Ferrari S. (2009). Macrophages are alternatively activated in patients with endometriosis and required for growth and vascularization of lesions in a mouse model of disease. Am. J. Pathol..

[27] Buvelot H., Jaquet V., Krause K.H. (2019). Mammalian NADPH Oxidases. Methods Mol. Biol..

[28] Kielland A., Blom T., Nandakumar K.S., Holmdahl R., Blomhoff R., Carlsen H. (2009). In vivo imaging of reactive oxygen and nitrogen species in inflammation using the luminescent probe L-012. Free Radic. Biol. Med..

[29] Wu M.H., Hsiao K.Y., Tsai S.J. (2019). Hypoxia: The force of endometriosis. J. Obstet. Gynaecol. Res..

[30] Kuhn H., Banthiya S., van Leyen K. (2015). Mammalian lipoxygenases and their biological relevance. Biochim. Biophys. Acta.

[31] Ivanov I., Kuhn H., Heydeck D. (2015). Structural and functional biology of arachidonic acid 15-lipoxygenase-1 (ALOX15). Gene.

[32] Cyrus T., Tang L.X., Rokach J., FitzGerald G.A., Pratico D. (2001). Lipid peroxidation and platelet activation in murine atherosclerosis. Circulation.

[33] Albadari N., Deng S., Li W. (2019). The transcriptional factors HIF-1 and HIF-2 and their novel inhibitors in cancer therapy. Expert Opin. Drug Discov..

[34] Morris G., Gevezova M., Sarafian V., Maes M. (2022). Redox regulation of the immune response. Cell. Mol. Immunol..

[35] Welsh S., Williams R., Kirkpatrick L., Paine-Murrieta G., Powis G. (2004). Antitumor activity and pharmacodynamic properties of PX-478, an inhibitor of hypoxia-inducible factor-1alpha. Mol. Cancer Ther..

[36] Schnurr K., Belkner J., Ursini F., Schewe T., Kuhn H. (1996). The selenoenzyme phospholipid hydroperoxide glutathione peroxidase controls the activity of the 15-lipoxygenase with complex substrates and preserves the specificity of the oxygenation products. J. Biol. Chem..

[37] Kakularam K.R., Karst F., Polamarasetty A., Ivanov I., Heydeck D., Kuhn H. (2022). Paralog- and ortholog-specificity of inhibitors of human and mouse lipoxygenase-isoforms. Biomed. Pharmacother..

[38] Adel S., Karst F., Gonzalez-Lafont A., Pekarova M., Saura P., Masgrau L., Lluch J.M., Stehling S., Horn T., Kuhn H. (2016). Evolutionary alteration of ALOX15 specificity optimizes the biosynthesis of antiinflammatory and proresolving lipoxins. Proc. Natl. Acad. Sci. USA.

[39] Macpherson G.R., Singh A.S., Bennett C.L., Venzon D.J., Liewehr D.J., Franks M.E., Dahut W.L., Kantoff P.W., Price D.K., Figg W.D. (2004). Genotyping and functional analysis of the D104N variant of human endostatin. Cancer Biol. Ther..

[40] Becker C.M., Rohwer N., Funakoshi T., Cramer T., Bernhardt W., Birsner A., Folkman J., D’Amato R.J. (2008). 2-methoxyestradiol inhibits hypoxia-inducible factor-1alpha and suppresses growth of lesions in a mouse model of endometriosis. Am. J. Pathol..

[41] Movafagh S., Crook S., Vo K. (2015). Regulation of hypoxia-inducible factor-1a by reactive oxygen species: New developments in an old debate. J. Cell. Biochem..

[42] Sobrido-Cameán D., Coulson B., Miller M., Oswald M.C.W., Pettini T., Bailey D.M.D., Baines R.A., Landgraf M. (2025). Mitochondrial ROS and HIF-1α signaling mediate synaptic plasticity in the critical period. PLoS Biol..

[43] Brunelle J.K., Bell E.L., Quesada N.M., Vercauteren K., Tiranti V., Zeviani M., Scarpulla R.C., Chandel N.S. (2005). Oxygen sensing requires mitochondrial ROS but not oxidative phosphorylation. Cell Metab..

[44] Guzy R.D., Hoyos B., Robin E., Chen H., Liu L., Mansfield K.D., Simon M.C., Hammerling U., Schumacker P.T. (2005). Mitochondrial complex III is required for hypoxia-induced ROS production and cellular oxygen sensing. Cell Metab..

[45] Khoufache K., Bazin S., Girard K., Guillemette J., Roy M.C., Verreault J.P., Al-Abed Y., Foster W., Akoum A. (2012). Macrophage migration inhibitory factor antagonist blocks the development of endometriosis in vivo. PLoS ONE.

[46] Keenan J.A., Chen T.T., Chadwell N.L., Torry D.S., Caudle M.R. (1994). Interferon-gamma (IFN-gamma) and interleukin-6 (IL-6) in peritoneal fluid and macrophage-conditioned media of women with endometriosis. Am. J. Reprod. Immunol..

[47] Akoum A., Kong J., Metz C., Beaumont M.C. (2002). Spontaneous and stimulated secretion of monocyte chemotactic protein-1 and macrophage migration inhibitory factor by peritoneal macrophages in women with and without endometriosis. Fertil. Steril..

[48] Haeggstrom J.Z., Funk C.D. (2011). Lipoxygenase and leukotriene pathways: Biochemistry, biology, and roles in disease. Chem. Rev..

[49] Snodgrass R.G., Brüne B. (2019). Regulation and Functions of 15-Lipoxygenases in Human Macrophages. Front. Pharmacol..

[50] Goteri G., Lucarini G., Montik N., Zizzi A., Stramazzotti D., Fabris G., Tranquilli A.L., Ciavattini A. (2009). Expression of vascular endothelial growth factor (VEGF), hypoxia inducible factor-1alpha (HIF-1alpha), and microvessel density in endometrial tissue in women with adenomyosis. Int. J. Gynecol. Pathol..

[51] Zhang T., Zhou J., Man G.C.W., Leung K.T., Liang B., Xiao B., Ma X., Huang S., Huang H., Hegde V.L. (2018). MDSCs drive the process of endometriosis by enhancing angiogenesis and are a new potential therapeutic target. Eur. J. Immunol..

[52] Vizzielli G., Cosentino F., Raimondo D., Turco L.C., Vargiu V., Iodice R., Mastronardi M., Mabrouk M., Scambia G., Seracchioli R. (2020). Real three-dimensional approach vs two-dimensional camera with and without real-time near-infrared imaging with indocyanine green for detection of endometriosis: A case-control study. Acta Obstet. Gynecol. Scand..

[53] Mosser D.M., Edwards J.P. (2008). Exploring the full spectrum of macrophage activation. Nat. Rev. Immunol..

[54] Radzun H.J., Parwaresch M.R. (1983). Differential immunohistochemical resolution of the human mononuclear phagocyte system. Cell. Immunol..

[55] Galdiero M.R., Garlanda C., Jaillon S., Marone G., Mantovani A. (2013). Tumor associated macrophages and neutrophils in tumor progression. J. Cell. Physiol..

[56] Shapouri-Moghaddam A., Mohammadian S., Vazini H., Taghadosi M., Esmaeili S.A., Mardani F., Seifi B., Mohammadi A., Afshari J.T., Sahebkar A. (2018). Macrophage plasticity, polarization, and function in health and disease. J. Cell. Physiol..

[57] Vallve-Juanico J., Baron C., Suarez-Salvador E., Castellvi J., Ballesteros A., Gil-Moreno A., Santamaria X. (2018). Lgr5 Does Not Vary Throughout the Menstrual Cycle in Endometriotic Human Eutopic Endometrium. Int. J. Mol. Sci..

[58] Poli-Neto O.B., Meola J., Rosa E.S.J.C., Tiezzi D. (2020). Transcriptome meta-analysis reveals differences of immune profile between eutopic endometrium from stage I-II and III-IV endometriosis independently of hormonal milieu. Sci. Rep..

[59] Kieran M.W., Kalluri R., Cho Y.J. (2012). The VEGF pathway in cancer and disease: Responses, resistance, and the path forward. Cold Spring Harb. Perspect. Med..

