# Supplementary material for: Oxidative Stress Mediated by Macrophages Promotes Angiogenesis and Early Development of Endometriosis
Source: Antioxidants (Basel). 2026 Jan 23;15(2):159. doi: 10.3390/antiox15020159 (PMC12937962; doi:10.3390/antiox15020159)
Supplement: Supplementary file 1 [file antioxidants-15-00159-s001.zip › antioxidants-4062865-supplementary.pdf]

Supplementary Table S1. Primary antibodies for semi-quantitative analysis

Markers	Concentration	Clonality	Isotype	Company
VEGF-A	1:50	Monoclonal	IgG	Abcam
F4/80	1:50	Monoclonal	IgG	Abcam
Neutrophils	1:50	Monoclonal	IgG2a	Abcam

VEGFA, vascular endothelial growth factor A

Supplementary Table S2. TaqMan primers sequence used for quantitative real-time analysis.

Gene	Taqman Probe	Amplicon Length
HIF-1α	Mm00468875_ml	88 bp
VEGF-A	Mm01281449ml	81 bp
CD31	Mm01242584_ml	71 bp
CD34	Mm00519283_ml	61 bp
GAPDH*	Mm99999915_gl	107 bp

HIF-1α, Hypoxic inducible factor-1alpha; VEGFA, vascular endothelial growth factor A; PECAM1 (CD31), platelet/endothelial cell adhesion molecule; CD34, CD34 antigen; GAPDH, glyceraldehyde-3-phosphate dehydrogenase

**Housekeeping gene*
